# Dermoscopic Interface Features in Melanoma–Seborrheic Keratosis Collision Tumors: A Scoping Review with an Illustrative Case Report on Wood’s Lamp Dermoscopy

**DOI:** 10.3390/diagnostics16081226

**Published:** 2026-04-20

**Authors:** Alexandre Raphael Meduri, Francesca Ambrogio, Lucia Lospalluti, Domenico Bonamonte, Giulia Ciccarese, Gerardo Cazzato, William Andrew Rosato, Paolo Romita, Mario Della Mura, Hugo Guillermou, Caterina Foti

**Affiliations:** 1 Section of Dermatology and Venereology, Department of Precision and Regenerative Medicine and Ionian Area (DiMePRe-J), University of Bari “Aldo Moro”, 70121 Bari, Italy; meduri.alexandre@gmail.com (A.R.M.); lucia.lospalluti@policlinico.ba.it (L.L.); domenico.bonamonte@uniba.it (D.B.); dr.williamrosato@gmail.com (W.A.R.); paolo.romita@uniba.it (P.R.); caterina.foti@uniba.it (C.F.); 2 Section of Dermatology, Department of Medical and Surgical Sciences, University of Foggia, 71121 Foggia, Italy; giulia.ciccarese@unifg.it; 3 Section of Molecular Pathology, Department of Precision and Regenerative Medicine and Ionian Area (DiMePRe-J), University of Bari “Aldo Moro”, 70121 Bari, Italy; gerardo.cazzato@uniba.it (G.C.); mariodellamura1@gmail.com (M.D.M.); 4 Department of Biostatistics, Epidemiology, Public Health and Innovation in Methodology, University of Nimes, 30000 Nimes, France; hugo.guillermou@chu-nimes.fr

**Keywords:** melanoma, seborrheic keratosis, collision tumors, dermoscopy, dermoscopic interface, diagnostic pitfalls, pigmented skin lesions, wood’s lamp dermoscopy, tumor microenvironment, interface pigmentation

## Abstract

**Background/Objectives:** Collision tumors between seborrheic keratosis and melanoma represent a well-known diagnostic pitfall, particularly when the benign keratinocytic component constitutes the predominant portion of the lesion. In such cases, melanoma-specific dermoscopic clues may be obscured by typical seborrheic keratosis patterns, leading to potential underestimation. The aim of this scoping review was to map and summarize the dermoscopic interface features reported in melanoma–seborrheic keratosis collision tumors. Secondary aims were to discuss diagnostic pitfalls, explore potential tumor microenvironment considerations, and assess the adjunctive role of Wood’s lamp-assisted dermoscopy. **Methods:** This review was conducted as a scoping review and reported according to the PRISMA-ScR guidelines using PubMed, Scopus, and Web of Science. Studies reporting histologically confirmed melanoma–seborrheic keratosis collision tumors with available dermoscopic documentation were included. Eligible articles consisted of case reports and case series. Dermoscopic features at the interface between seborrheic keratosis and melanoma were qualitatively synthesized. **Results:** Five studies describing five melanoma-seborrheic keratosis collision tumors met the inclusion criteria. In all cases, the seborrheic keratosis component was dermoscopically recognizable. Asymmetric interface-related hyperpigmentation was consistently observed in collisions involving pigmented melanomas, whereas it was absent in the single reported case of hypopigmented melanoma. **Conclusions:** Asymmetric interface-related hyperpigmentation within seborrheic keratosis is a recurrent dermoscopic finding in melanoma–seborrheic keratosis collision tumors and could be considered a monitoring clue rather than a melanoma-specific diagnostic criterion. Given the dynamic nature of melanoma growth, longitudinal assessment of the dermoscopic interface may be particularly informative. Adjunctive techniques, including Wood’s lamp-assisted dermoscopy, may support interface-focused evaluation in selected equivocal cases.

## 1. Introduction

Dermoscopy represents a cornerstone in the clinical evaluation and early diagnosis of cutaneous tumors, significantly increasing diagnostic accuracy compared with naked-eye examination and contributing to earlier detection of cutaneous melanoma, one of the most aggressive and potentially lethal skin cancers [1,2]. Since its introduction into routine dermatologic practice in the 1990s, dermoscopy has profoundly changed the assessment of pigmented lesions by enabling visualization of subsurface morphologic structures not appreciable to the unaided eye.

From a dermoscopic perspective, melanoma typically displays an asymmetric distribution of colors and structures. Additional melanoma-associated dermoscopic features, such as irregular pseudopods, may also represent early clues of melanocytic malignancy, particularly in small or early-stage melanomas. Common dermoscopic hallmarks include an atypical pigment network, irregularly distributed dots and globules, uneven streaks or lines, and poorly defined areas of blotchy or hyperpigmented pigmentation. Additional features such as shiny white streaks, regression-related structures, a blue–white veil, and polymorphous vascular patterns are frequently observed, particularly in invasive melanoma [3].

The most recent European consensus-based interdisciplinary guideline for melanoma diagnosis and management stated that dermoscopy supports the clinical evaluation of suspected melanoma, while histopathologic examination remains the diagnostic gold standard [3]. Dermoscopic examination should not be limited to lesions that appear clinically suspicious, as it may disclose early morphological asymmetry and melanoma-related features at a stage when clinical assessment alone is still non-contributory.

In high-risk patients, sequential digital dermoscopy combined with total-body photography further improves the detection of early-stage melanoma [3].

When available, reflectance confocal microscopy may provide additional diagnostic support in selected challenging cases, enhancing diagnostic confidence and specificity in equivocal melanocytic lesions with indeterminate dermoscopic features [3,4].

Despite the technological advances, melanoma remains a diagnostically challenging entity due to its marked clinical and dermoscopic heterogeneity [3]. This difficulty is especially evident when melanoma collides with a benign lesion, as mixed or overlapping morphologic features may obscure melanoma-specific clues and hinder accurate dermoscopic interpretation [5].

Collision tumors, defined as the coexistence of two histologically distinct neoplasms maintaining distinct boundaries within the same lesion, may be difficult to diagnose even by expert dermoscopists exemplifying this diagnostic scenario [5].

Among collision tumors, melanoma–seborrheic keratosis combinations represent a particularly challenging diagnostic scenario. Seborrheic keratoses are common benign keratinocytic neoplasms characterized by prominent keratin-related dermoscopic structures, including milia-like cysts, comedo-like openings, fissures, and cerebriform patterns [6]. These reassuring features may dominate the overall dermoscopic appearance of the lesion, potentially masking or diluting melanoma-associated criteria and increasing the risk of diagnostic underestimation or delay.

A subset of melanoma–seborrheic keratosis collision tumors involves lentigo maligna and lentigo maligna melanoma. Lentigo maligna represents a melanoma in situ subtype that typically arises on chronically sun-damaged skin of elderly patients, particularly on the head and neck. Dermoscopically, lentigo maligna is characterized by subtle and often incomplete melanocytic features, including asymmetric pigmented follicular openings, annular–granular structures, rhomboidal patterns, and irregular perifollicular pigmentation [1,7]. These features may be focal and discontinuous, rendering lentigo maligna particularly difficult to recognize, especially when coexisting with keratinocytic lesions, and making it especially prone to diagnostic masking in collision settings.

In this context, increasing attention has been directed toward the spatial distribution of dermoscopic features within complex lesions. In collision tumors, particular diagnostic relevance may lie in the interface between the keratinocytic and melanocytic components, defined as the area of contiguity where morphologic features of both components may coexist or juxtapose within the same lesion. Careful evaluation of asymmetries or incongruent pigmentation patterns at this interface may provide indirect clues to an underlying melanocytic proliferation in lesions otherwise suggestive of seborrheic keratosis.

To further support this diagnostic process, complementary dermoscopic techniques have been proposed. Wood’s lamp-assisted dermoscopy, employing long-wave ultraviolet radiation at approximately 365 nm, enhances the visualization of superficial keratin-related structures [8]. By facilitating more accurate delineation of the keratinocytic component, this adjunctive approach may indirectly facilitate recognition of lesion areas warranting closer dermoscopic evaluation for melanoma, although its role in collision tumors has not yet been systematically analyzed.

The aim of this scoping review was to map the clinical and dermoscopic features of lentigo maligna and melanoma–seborrheic keratosis collision tumors with available dermoscopic documentation, with particular focus on recurrent interface-related patterns and potential diagnostic clues, and to explore the adjunctive role of Wood’s lamp-assisted dermoscopy in improving recognition of these diagnostically challenging entities.

## 2. Materials and Methods

### 2.1. Study Design

This study was conducted as a scoping review of the literature on the dermoscopic and histologic features of melanoma–seborrheic keratosis collision tumors. This review was conducted as a scoping review and reported according to the Preferred Reporting Items for Systematic Reviews and Meta-Analyses extension for Scoping Reviews (PRISMA-ScR) guidelines [9,10].

The research question was structured according to a PCC (Population–Concept–Context) framework commonly used in scoping reviews. The Population consisted of patients with melanoma–seborrheic keratosis collision tumors, the Concept referred to dermoscopic interface features, and the Context included published reports with dermoscopic documentation of these lesions. When full access to articles was missing, study authors were contacted to obtain the articles.

Similarly, authors were also contacted when outcome data were not reported in full to acquire additional outcomes data.

This scoping review was not registered in PROSPERO.

### 2.2. Search Strategy

A comprehensive literature search was performed in the electronic databases PubMed, Scopus, and Web of Science to identify studies reporting melanoma–seborrheic keratosis collision tumors with available dermoscopic documentation. No date restrictions were applied; the search included all records available in the selected databases up to 26 December 2025.

Title/abstract screening and full-text assessment were performed independently by two reviewers (A.R.M and F.A), with discrepancies resolved by consensus.

Reference lists of all included articles were manually screened to identify additional potentially relevant reports.

*Primary database search*:

The primary search was conducted using predefined keywords and Boolean operators:

(melanoma OR “lentigo maligna”) AND (“seborrheic keratosis” OR “seborrhoeic keratosis”) AND (collision OR combined OR coexist OR associated).

To ensure comprehensive retrieval of potentially relevant reports, a second, independent search was performed using broader collision-related terms: “collision skin lesion” AND (dermoscopy OR dermatoscopy).

Grey literature sources and conference abstracts were not systematically searched, which may introduce a potential publication bias.

### 2.3. Eligibility Criteria

Studies were considered eligible for inclusion if they met all of the following criteria:

1. Reported cases of histologically confirmed melanoma–seborrheic keratosis collision tumors, with both components clearly identified on histopathologic examination.
2. Included dermoscopic documentation (clinical images or detailed dermoscopic descriptions) allowing assessment of dermoscopic features of either or both colliding components.
3. Were published as original articles, case reports, or case series.
4. Were available as full-text articles.
5. Only articles with full text written in English were included due to resource limitations and the inability to reliably translate non-English full texts, which may introduce language bias.

Studies were excluded if they:

1. Did not describe a true collision between melanoma and seborrheic keratosis.
2. Reported combined or associated skin lesions without histopathologic confirmation of both tumor components.
3. Lacked dermoscopic images or sufficient dermoscopic description of the skin lesion.
4. Represented duplicate publications of previously reported cases.

No restrictions were applied regarding patient age, sex, anatomical location of the lesion, or melanoma subtype, in order to capture the full spectrum of reported collision tumors.

### 2.4. Data Extraction

Data were extracted from all included studies using a standardized data collection form developed a priori. Given the descriptive nature of the available literature, data extraction focused on dermoscopic and interface-related features rather than patient-level outcomes.

Data extraction was performed independently by two reviewers (A.R.M and F.A) using a standardized data collection form.

For the purpose of this review, the dermoscopic interface was defined as the area of contiguity between the seborrheic keratosis and melanoma components, as identified by the coexistence or juxtaposition of their respective dermoscopic structures within the same lesion.

Interface-related features were assessed to explore whether the proximity of melanoma could influence the dermoscopic appearance of the adjacent seborrheic keratosis component, potentially resulting in focal pigmentation asymmetry.

The following variables were extracted when available:

- Melanoma pigmentation (pigmented: yes/no).
- Dermoscopic morphology of the seborrheic keratosis component (flat/reticular-like, cerebriform/acanthotic, mixed, or unclear).
- Collision configuration, categorized according to the visually dominant component (seborrheic keratosis–dominant, melanoma-dominant, or approximately equal components).
- Presence of dermoscopic interface hyperpigmentation within the seborrheic keratosis component.
- Asymmetry of seborrheic keratosis pigmentation, comparing the melanoma-adjacent portion with the non-adjacent portion.

Other dermoscopic melanoma-specific criteria were inconsistently reported across the included studies and therefore could not be systematically extracted or analyzed.

In studies reporting multiple collision tumors, only cases involving melanoma and seborrheic keratosis with available dermoscopic documentation of the interface were included in the analysis.

Extracted data were synthesized qualitatively and summarized descriptively to identify recurrent dermoscopic interface features and potential diagnostic clues in melanoma–seborrheic keratosis collision tumors.

### 2.5. Quality Assessment

Given that all included studies were case reports or case series, a formal quantitative risk-of-bias assessment was not appropriate. Instead, study quality was evaluated using a simplified critical appraisal approach, focusing on:

- clarity and completeness of case description.
- histopathologic confirmation of both tumor components.
- availability and quality of dermoscopic documentation.

This pragmatic approach was adopted to ensure transparency while acknowledging the intrinsic limitations of descriptive evidence. Detailed quality appraisal results are provided in the Supplementary Material.

### 2.6. Data Synthesis

Results were synthesized using a descriptive qualitative approach. Due the exclusively descriptive nature of the available evidence, no quantitative synthesis or meta-analysis was performed.

Dermoscopic findings were summarized narratively and organized into tabular form to facilitate comparison across studies and to highlight recurrent interface-related features. Particular emphasis was placed on the distribution of pigmentation within the seborrheic keratosis component and its relationship to the melanoma interface.

No subgroup or sensitivity analyses were conducted.

### 2.7. Illustrative Case Report

In addition to the scoping review, an illustrative clinical case of a melanoma–seborrheic keratosis collision tumor was included. The case was observed in routine clinical practice and was not identified through the systematic literature search. Dermoscopic examination was performed using polarized light to reduce surface reflection and was complemented by Wood’s lamp-assisted dermoscopy. An initial diagnostic biopsy was performed, while the definitive histopathologic diagnosis was established on the excisional specimen.

To our knowledge, this case represents the sixth reported melanoma–seborrheic keratosis collision tumor with dermoscopic documentation in the literature.

The case is presented for illustrative and educational purposes only and did not contribute to the systematic evidence synthesis.

## 3. Results

### 3.1. Study Selection

All records identified through the primary database search were merged, and duplicate entries were removed prior to screening. Titles and abstracts of the remaining records were screened to assess potential relevance. Articles deemed potentially eligible were subsequently retrieved in full text and evaluated for inclusion based on the predefined eligibility criteria. Following title and abstract screening of the primary search results, 20 articles were considered potentially eligible and selected for full-text retrieval. Two articles could not be retrieved, and one article was excluded as a duplicate full-text publication. Consequently, 17 full-text articles were assessed for eligibility, of which four fulfilled all inclusion criteria and were included in the qualitative synthesis. Among the 13 excluded full-text articles, 5 did not report true melanoma–seborrheic keratosis collision tumors, and 8 lacked dermoscopic documentation. In addition, records identified through the supplementary database search were screened using the same eligibility criteria. Of the articles retrieved for full-text evaluation from this supplementary search, one study met all inclusion criteria and was included in the qualitative synthesis. Overall, the combined search strategies yielded 485 records (127 from PubMed, 13 from Scopus, and 345 from Web of Science). After merging all retrieved records and removing 119 duplicates, 366 unique articles were identified for title and abstract screening.

**Figure 1:**
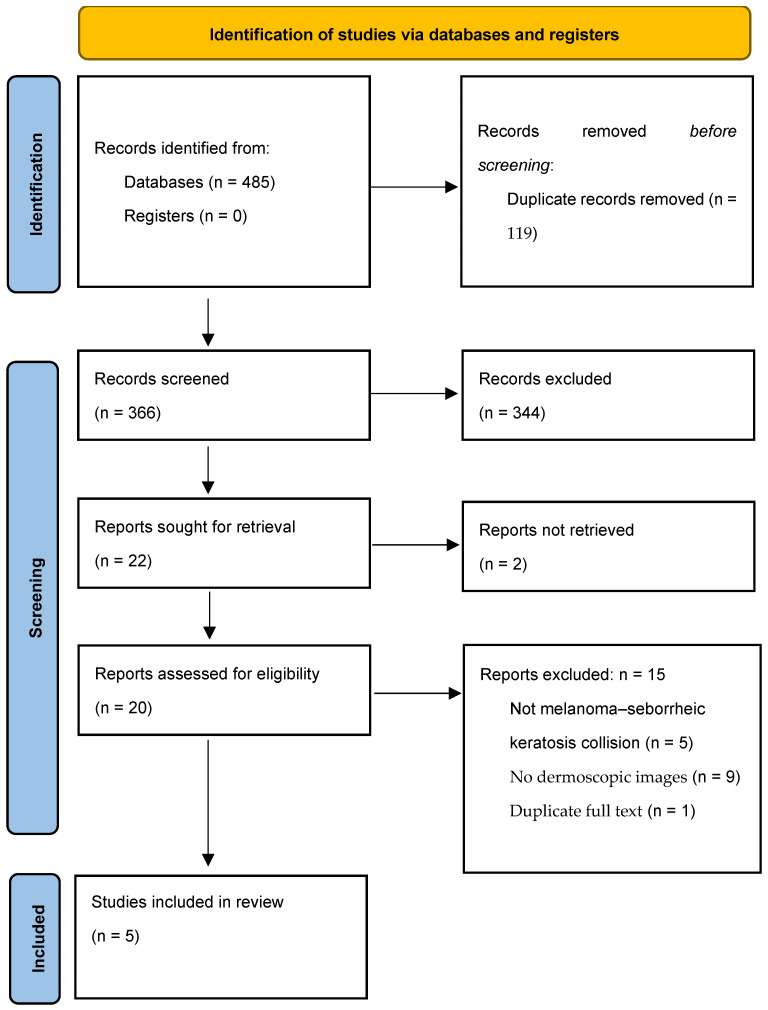
PRISMA flow diagram illustrating the study selection process for this scoping review of melanoma–seborrheic keratosis collision tumors.

Overall, five studies were included in the qualitative synthesis [11,12,13,14,15]. The study selection process and reasons for exclusion are summarized in the PRISMA flow diagram (Figure 1).

### 3.2. Study Characteristics

A total of five studies describing five melanoma-seborrheic keratosis collision tumors met the inclusion criteria and were included in the scoping review. All included studies were case reports or case series describing histologically confirmed melanoma–seborrheic keratosis collision tumors with available dermoscopic images.

### 3.3. Dermoscopic Interface Features

Dermoscopic interface features extracted from the included studies are summarized in Table 1.

The melanoma component was pigmented in 4 of 5 studies, whereas 1 study described a hypopigmented melanoma. In all cases, the seborrheic keratosis component was clearly identifiable on dermoscopic examination.

Asymmetric pigmentation of the seborrheic keratosis component was observed in all included cases (5/5). Interface-related hyperpigmentation of the seborrheic keratosis component was present in 4 of 5 cases and was consistently localized to the portion adjacent to the melanoma. In the remaining case, interface-related hyperpigmentation was not observed, and the melanoma component was hypopigmented.

It should be noted that most reported cases involved acanthotic seborrheic keratoses, characterized by keratin-related dermoscopic structures including cerebriform/acanthotic patterns, verrucous structures with comedo-like openings and milia-like cysts. These features may be less evident or absent in thin flat seborrheic keratoses, which may therefore present greater diagnostic difficulty in collision settings.

Regarding spatial configuration, collision patterns were categorized as seborrheic keratosis–dominant in 2 cases, approximately equal in 2 cases, and melanoma-dominant in 1 case.

### 3.4. Illustrative Clinical Case

An 80-year-old woman underwent a routine dermatologic examination as part of a nevus surveillance program. During the visit, a previously unnoticed pigmented lesion was identified on the right cheek. The patient did not report recent changes in size or associated symptoms.

At the initial examination, dermoscopy revealed a complex lesion with morphologically heterogeneous features, including a melanocytic component showing atypical characteristics that raised suspicion for a melanocytic proliferation. Based on these findings, a diagnostic biopsy was performed, targeting the irregularly pigmented melanocytic area (yellow arrow). Histopathologic examination of the biopsy specimen revealed lentigo maligna.

**Figure 2:**
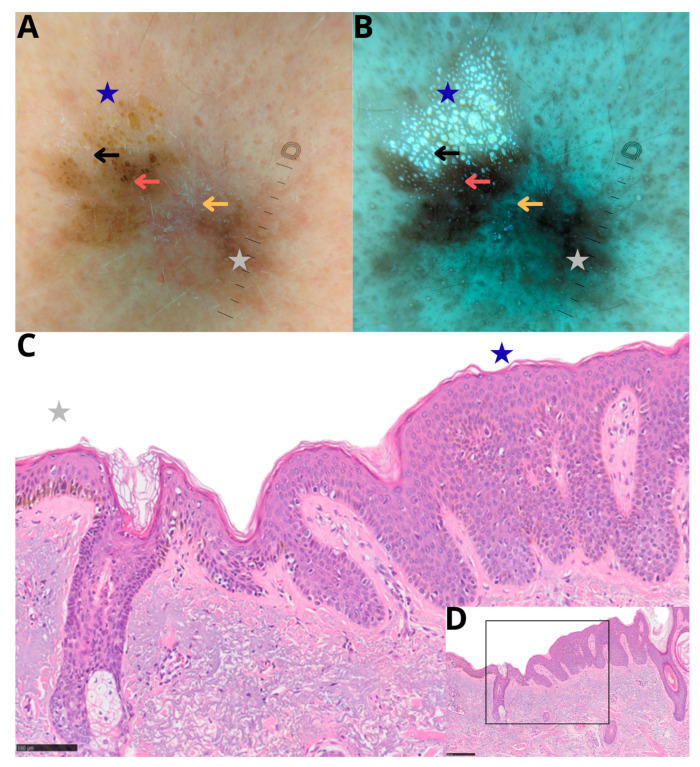
Dermoscopic and histopathologic correlation in a melanoma–seborrheic keratosis collision tumor. (**A**,**B**) Polarized dermoscopy and Wood’s lamp-assisted dermoscopy show a collision lesion composed of a seborrheic keratosis component (blue star), characterized by keratin-related structures, adjacent to an irregularly pigmented melanocytic component (grey star). The seborrheic keratosis exhibits asymmetric darkening at the interface with the melanocytic area (red arrow). Under ultraviolet illumination, keratin-rich structures become more conspicuous (black arrow), while pigmented keratin pearls at the interface show reduced visibility (red arrow). A small atrophic area is visible (yellow arrow). (**C**) Histopathologic examination at intermediate magnification (H&E, scale bar: 100 microm) demonstrates an acanthotic seborrheic keratosis (blue star) adjacent to a lentiginous melanocytic proliferation consistent with lentigo maligna melanoma (grey star). Sparse melanin pigment can be seen throughout the epidermis and at the interface between the two lesions. (**D**) Low-power overview of the same histologic section (H&E, scale bar: 250 microm) illustrates the spatial relationship between the seborrheic keratosis and the melanocytic component and indicates the area shown at higher magnification in (**C**).

Following the histopathologic diagnosis and prior to definitive surgical excision, dermoscopic documentation of the lesion was obtained for illustrative purposes (Figure 2A). Polarized dermoscopy demonstrated two contiguous but morphologically distinct components (Figure 2A). One portion showed typical dermoscopic features of a seborrheic keratosis (blue star), whereas the adjacent area exhibited irregular melanocytic features (grey star), including asymmetric pigmentation, ill-defined borders, and irregular hyperpigmentation of follicular openings.

At the same time point, Wood’s lamp-assisted dermoscopy was performed as an adjunctive imaging technique (Figure 2B). Under ultraviolet illumination, keratin-rich structures within the seborrheic keratosis component (black arrow) became more conspicuous, facilitating clearer visual differentiation between the keratinocytic and melanocytic components and allowing a more accurate appreciation of interface-related pigmentation differences (red arrow).

The lesion was subsequently excised in toto. Final histopathologic examination of the excisional specimen led to reclassification of the lesion as lentigo maligna melanoma, revealing a melanoma–seborrheic keratosis collision tumor (Figure 2D), composed of an acanthotic seborrheic keratosis adjacent to a lentiginous melanocytic proliferation with a focal invasive component. The invasive component measured 0.3 mm in Breslow thickness.

The case is presented for illustrative purposes to highlight the diagnostic challenges posed by melanoma–seborrheic keratosis collision tumors and to emphasize the potential value of interface-focused dermoscopic assessment and adjunctive imaging techniques in selected complex lesions. Based on the currently available literature, this case represents the sixth reported melanoma–seborrheic keratosis collision tumor.

These observations derive from a single illustrative case and should therefore be considered exploratory and hypothesis-generating.

## 4. Discussion

### 4.1. Dermoscopic Interface Features in Melanoma–Seborrheic Keratosis Collision Tumors

This scoping review suggests a recurrent dermoscopic pattern at the interface between seborrheic keratosis and melanoma. Across the included cases, asymmetric pigmentation of the seborrheic keratosis component was consistently observed, while interface-related hyperpigmentation was present in the majority of cases.

These findings suggest that the interface between the keratinocytic and melanocytic components may represent a relevant area for dermoscopic assessment in collision tumors. In this context, we propose a speculative conceptual interpretation in which the seborrheic keratosis component may act as a “keratinocytic sponge”, retaining or accentuating pigment originating from the adjacent melanocytic proliferation.

From a structural perspective, seborrheic keratoses are characterized by hyperkeratosis, acanthosis, and an irregular epidermal architecture, which may facilitate accumulation or visual enhancement of melanin or pigmented material at the interface. This phenomenon could potentially account for the localized darkening of the seborrheic keratosis component adjacent to melanoma, without implying direct melanocytic infiltration or malignant transformation of the keratinocytic tissue.

Importantly, the absence of interface-related hyperpigmentation in the case involving a hypopigmented melanoma supports this interpretation, suggesting that visible pigmentation of the melanocytic component may be a prerequisite for the development of interface-associated darkening within the seborrheic keratosis component.

Taken together, these observations may support the view that asymmetric pigmentation within a seborrheic keratosis in the context of a collision tumor should be interpreted as a nonspecific monitoring clue rather than a melanoma-specific diagnostic criterion. The “keratinocytic sponge” concept should be regarded as a speculative interpretative model to explain interface-related dermoscopic changes, while underscoring the need for careful evaluation of adjacent melanocytic structures.

### 4.2. Diagnostic Pitfalls in Melanoma–Seborrheic Keratosis Collision Tumors

Melanoma–seborrheic keratosis collision tumors represent a well-recognized diagnostic challenge in daily dermatologic practice [15]. This difficulty arises not only from the coexistence of benign and malignant components within the same lesion, but also from the frequent subtlety and incomplete expression of melanoma-related dermoscopic features in this context.

In the reviewed cases, the melanoma component did not consistently represent the predominant portion of the lesion. When melanoma occupies a limited or non-dominant area within a complex lesion, this spatial distribution may induce diagnostic underestimation, particularly in the setting of early-stage melanoma, in which not all classical dermoscopic criteria of malignancy are necessarily present or fully developed [1,7]. As a consequence, melanoma-associated features may appear focal, discontinuous, or weakly contrasted, increasing the likelihood that they remain undetected during routine dermoscopic examination, especially when embedded within a morphologically complex lesion.

Seborrheic keratoses display a wide spectrum of keratin-related dermoscopic structures, including cerebriform architecture, comedo-like openings, milia-like cysts, and fissures and ridges. These features are generally regarded as reassuring and may strongly influence diagnostic reasoning [16,17]. In collision tumors, however, their presence may complicate lesion assessment by diverting attention toward the benign keratinocytic component, while subtle melanocytic features located in adjacent areas receive less scrutiny. Importantly, the diagnostic challenge does not depend solely on the visual dominance of the seborrheic keratosis component, but rather on the relative inconspicuousness and limited spatial extent of the melanoma component.

An additional diagnostic challenge may arise when lentigo maligna collides with a thin flat seborrheic keratosis, which may also appear as a pigmented macule lacking prominent keratin-related structures. In these cases, classic dermoscopic features such as milia-like cysts or comedo-like openings may be absent, making recognition of the keratinocytic component more difficult.

In this context, reliance on global pattern recognition or on the identification of typical seborrheic keratosis criteria alone may be insufficient. Instead, careful intra-lesional comparison becomes essential. Evaluation of dermoscopic asymmetry, differences in pigmentation intensity, and variation in structure distribution between melanoma-adjacent and non-adjacent areas of the same lesion may provide indirect clues to the presence of a melanocytic proliferation that warrants closer evaluation.

An additional layer of complexity is introduced by the often indolent or slowly progressive clinical behavior of collision lesions. When a melanocytic proliferation collides with a long-standing seborrheic keratosis, changes in pigmentation or asymmetry may evolve gradually and remain clinically unapparent for extended periods [18]. In such cases, short-term dermoscopic monitoring and careful assessment of temporal changes, particularly at the interface between lesion components, may represent a useful complementary strategy to single time-point evaluation.

A further clinically relevant pitfall concerns the increasing use of destructive or cosmetic procedures, such as laser ablation, for the treatment of seborrheic keratoses [19]. In the presence of an unrecognized melanocytic component within or adjacent to a seborrheic keratosis, such interventions may delay melanoma diagnosis or result in partial lesion removal without histopathologic assessment. For this reason, particular caution is warranted when evaluating seborrheic keratoses that exhibit focal or asymmetric pigmentation.

Color heterogeneity within seborrheic keratoses is frequently influenced by uneven hyperkeratosis and surface architecture, which can create optical variations unrelated to melanocytic activity. From a conceptual standpoint, it may therefore be useful to consider the relative contribution of hyperkeratosis versus true melanocytic pigmentation to observed color asymmetry. Reduction in surface hyperkeratosis may theoretically attenuate pigmentation irregularities driven predominantly by keratin-related optical effects, whereas persistence of focal pigmentation asymmetry despite reduction in hyperkeratosis could raise suspicion for an underlying or adjacent melanocytic proliferation. This hypothesis-generating interpretative framework should not be regarded as a diagnostic test, but rather as a tool to highlight lesions that warrant closer dermoscopic evaluation, histopathologic assessment, or avoidance of destructive treatment modalities.

Overall, melanoma–seborrheic keratosis collision tumors exemplify how early melanoma may evade detection when malignant features are incomplete, focal, and embedded within a complex lesion architecture. Awareness of these diagnostic pitfalls underscores the importance of interface-focused assessment, careful intra-lesional comparison, consideration of lesion evolution over time, and a cautious approach to destructive treatments in the presence of atypical pigmentation patterns.

### 4.3. Adjunctive Imaging Techniques in Melanoma–Seborrheic Keratosis Collision Tumors

Within the limits of the available literature, Wood’s lamp-assisted dermoscopy has not previously been reported in the evaluation of melanoma–seborrheic keratosis collision tumors. The illustrative case presented herein therefore provides an initial descriptive example of its potential application in this specific diagnostic context.

Given the diagnostic challenges posed by melanoma–seborrheic keratosis collision tumors, adjunctive imaging techniques may play a supportive role when conventional dermoscopic assessment yields equivocal findings. Situations characterized by focal melanocytic features, asymmetric pigmentation at the interface, or incomplete expression of melanoma-specific criteria may benefit from additional non-invasive evaluation.

Reflectance confocal microscopy (RCM) has been shown to improve the detection of melanocytic proliferations in complex or collision lesions by enabling in vivo visualization of cellular and architectural features beyond the resolution of dermoscopy [20]. In the setting of melanoma–seborrheic keratosis collision tumors, RCM may facilitate identification of atypical melanocytes at the dermoepidermal junction or within adnexal structures, even when the overlying keratinocytic component displays prominent benign features. However, despite its diagnostic value, the use of RCM remains limited by availability, cost, and the need for specialized expertise, restricting its routine application to selected referral centers [4].

These limitations highlight the need for additional, widely accessible tools that may complement dermoscopic evaluation in everyday clinical practice. In this context, techniques capable of enhancing contrast between epidermal components or facilitating separation of overlapping structures may be particularly useful in collision lesions, where diagnostic uncertainty often arises from the coexistence of morphologically distinct tissues within the same lesion.

Wood’s lamp-assisted dermoscopy represents one such adjunctive approach [21]. Unlike advanced imaging modalities such as reflectance confocal microscopy, this technique can be performed using portable, handheld devices and at substantially lower cost, making it more accessible in routine clinical practice; however, its diagnostic value in melanoma–seborrheic keratosis collision tumors remains exploratory and requires validation in larger studies. In recent years, the integration of long-wave ultraviolet illumination into modern dermatoscopes has facilitated renewed clinical interest in this modality. In particular, the introduction of hybrid devices incorporating Wood’s lamp functionality into standard dermoscopic examination, has enabled seamless switching between visible-light and ultraviolet-assisted dermoscopy within a single, pocket-sized instrument.

This technological development appears to have contributed to a growing body of recent literature exploring the potential applications of ultraviolet-assisted dermoscopic evaluation in pigmented lesions. Rather than providing cellular-level detail, Wood’s lamp-assisted dermoscopy supports lesion assessment by facilitating visual differentiation between coexisting epidermal components, particularly in complex or equivocal cases. Its potential role in melanoma–seborrheic keratosis collision tumors has not yet been systematically evaluated; however, its simplicity, portability, and ease of integration into routine workflows make it an appealing problem-solving tool in selected equivocal cases, particularly when more advanced imaging techniques are unavailable or impractical.

### 4.4. Wood’s Lamp-Assisted Dermoscopy: Optical Basis and Clinical Implications in Collision Tumors

Wood’s lamp-assisted dermoscopy provides a complementary optical perspective that may be useful in the evaluation of complex pigmented lesions, including melanoma–seborrheic keratosis collision tumors. By emitting long-wave ultraviolet radiation in the UVA spectrum (approximately 365 nm), this technique primarily interacts with superficial epidermal structures, with limited penetration into deeper skin layers [8,22]. As a result, it emphasizes epidermal contrast and may provide information that differs from conventional visible-light dermoscopy [23].

Under ultraviolet illumination, keratin-rich structures often appear brighter due to light scattering and fluorescence phenomena related to compact keratin and superficial epidermal components. In contrast, melanin strongly absorbs ultraviolet radiation and does not fluoresce, resulting in melanocytic pigmentation appearing darker and more sharply demarcated. This differential optical behavior increases contrast between epidermal components and may facilitate visual separation of coexisting lesion structures.

Another potentially useful dermoscopic clue in melanoma detection is variation in surface micromorphology compared with surrounding skin or adjacent lesions. In collision tumors, such discordance may be partially masked by the keratinocytic component, but techniques enhancing optical contrast, such as Wood’s lamp-assisted dermoscopy, could potentially facilitate recognition of these subtle differences. Future studies may explore this aspect further.

In melanoma–seborrheic keratosis collision tumors, enhanced contrast may translate into clearer delineation of lesion borders and the interface between keratinocytic and melanocytic components, as illustrated in the presented case. Under Wood’s lamp illumination, keratin-related structures that contribute to pigmentation under visible light may become less conspicuous, whereas areas of true melanocytic pigmentation remain evident due to ultraviolet absorption. This optical behavior may reduce keratin-related visual noise and allow more accurate appreciation of pigmentation distribution and lesion contours [24].

These properties may be particularly useful during conservative “wait-and-see” management, facilitating longitudinal assessment of subtle changes in pigmentation extent, asymmetry, or interface configuration over time. From a clinical perspective, Wood’s lamp-assisted dermoscopy should be regarded as a complementary, problem-solving tool rather than a standalone diagnostic modality. Although its diagnostic capabilities in differentiating melanocytic proliferations are still being explored, current evidence supports its role as an adjunct to conventional dermoscopy rather than a replacement [25].

Overall, while the role of Wood’s lamp-assisted dermoscopy in melanoma–seborrheic keratosis collision tumors remains exploratory, its integration into a multimodal diagnostic strategy may help mitigate some of the diagnostic challenges posed by complex collision lesions. Further studies are warranted toclarify its diagnostic contribution and optimal integration with conventional dermoscopy and other non-invasive imaging techniques.

### 4.5. Seborrheic Keratosis as a Permissive Melanocytic Microenvironment

Beyond optical and diagnostic considerations, the recurrent interface features observed in melanoma–seborrheic keratosis collision tumors raise the question of whether seborrheic keratosis may represent a biologically permissive microenvironment for melanocytic proliferation [26].

Although seborrheic keratoses are clinically benign and lack intrinsic malignant potential, they are now recognized as true clonal keratinocytic neoplasms characterized by recurrent activating mutations, most commonly involving FGFR3 and PIK3CA [27,28,29,30]. Despite these oncogenic alterations, seborrheic keratoses maintain a stable phenotype, likely due to preserved keratinocyte differentiation programs that counterbalance proliferative signaling [28,30]. These observations suggests that seborrheic keratoses are metabolically and signaling-active lesions rather than inert epidermal proliferations [27,28].

Melanocyte behavior within the epidermis is tightly regulated by the surrounding microenvironment through a complex network of keratinocyte-derived paracrine factors [31,32]. Keratinocytes modulate melanocyte survival, proliferation, and pigmentation via mediators such as stem cell factor (SCF), endothelin-1 (EDN1), α-melanocyte–stimulating hormone, hepatocyte growth factor, and pro-inflammatory cytokines [31]. Melanocytes express the corresponding receptors, including c-KIT, EDNRB, and MC1R, allowing their activity to be influenced by local epidermal signals even in the absence of activating mutations [31,32].

Within this framework, seborrheic keratoses may generate a localized epidermal niche enriched in melanocyte-modulating signals, potentially facilitating melanocytic persistence or focal activation at the lesion interface [26]. SCF–c-KIT signaling has been implicated in melanocyte survival and activation and appears particularly relevant in melanocytic proliferations arising on chronically sun-damaged skin, including lentiginous melanomas [33]. Endothelin-1-mediated signaling may further contribute to melanocyte stimulation within keratinocyte-rich environments [31,32].

Localized microenvironmental modulation may result in spatially heterogeneous melanocytic activity, potentially explaining why pigmentary changes are preferentially observed at the interface between seborrheic keratosis and the melanocytic component rather than uniformly across the lesion [26]. This focal biological activation could account for the dermoscopic observation of asymmetric interface pigmentation described in the present review.

While this hypothesis remains speculative, it provides a conceptual framework linking keratinocyte signaling, melanocytic behavior and the dermoscopic dynamics observed in collision tumors [26].

Although no causal relationship can be inferred from the available clinical evidence, this biologically permissive context provides a plausible explanation for interface-related pigmentation changes observed dermoscopically in seborrheic keratosis-associated collision tumors [31,32].

Importantly, similar microenvironmental interactions could theoretically influence not only melanoma but also benign melanocytic proliferations, such as nevi, supporting the interpretation of interface-related pigmentation changes as a dynamic monitoring clue rather than a melanoma-specific marker [26,31].

Taken together, these considerations support an interpretative model in which interface-related pigmentation changes reflect the convergence of optical masking, biological interaction, and temporal evolution, reinforcing their potential value as a clue for targeted monitoring rather than immediate diagnostic categorization [26].

### 4.6. Clinical Considerations and Monitoring Perspectives

Within the limitations of the available evidence, the findings of this scoping review suggest that seborrheic keratosis-associated pigmented lesions may require a more nuanced clinical evaluation than traditionally assumed. While typical seborrheic keratosis criteria are often reassuring, collision tumors highlight scenarios in which subtle melanocytic proliferations may coexist with prominent keratinocytic structures.

In this context, asymmetric pigmentation within a seborrheic keratosis—particularly when localized to the interface with another structural component—may represent a potential monitoring signal rather than a standalone diagnostic criterion. During longitudinal dermoscopic follow-up, changes in pigmentation distribution or extension at the interface may warrant closer evaluation, especially when other melanoma-specific features are incomplete or absent.

The present case illustrates how different dermoscopic modalities may offer complementary perspectives in complex lesions. Conventional and polarized dermoscopy support assessment of pigment distribution and melanocytic features, whereas Wood’s lamp-assisted dermoscopy may contribute by enhancing epidermal contrast and delineating keratinocytic components. Together, these approaches may help identify lesion areas that deviate from typical seborrheic keratosis morphology and therefore merit increased diagnostic attention.

From a practical standpoint, these considerations may be particularly relevant in selected clinical scenarios, such as lesions located on chronically sun-damaged skin or lesions with heterogeneous dermoscopic patterns. Although adjunctive techniques do not replace histopathologic evaluation, they may assist clinicians in refining judgment regarding follow-up strategies, biopsy targeting, or timing of excision.

### 4.7. Limitations

This scoping review has some limitations, mainly related to the rarity of melanoma–seborrheic keratosis collision tumors and the exclusively descriptive nature of the available literature, which consists of case reports and small case series and does not allow quantitative synthesis or assessment of diagnostic accuracy metrics. Heterogeneity in dermoscopic documentation and reporting across studies may also have influenced feature interpretation. This review was not registered in PROSPERO, which may limit methodological transparency. In addition, only articles written in English were included, which may introduce language bias.

Despite these limitations, this study has several relevant strengths. It proposes a previously underrecognized dermoscopic interface-related pigmentation pattern as a potential novel descriptive sign in melanoma–seborrheic keratosis collision tumors, generating a testable hypothesis that warrants confirmation in larger, prospective studies. In addition, the exploratory use of Wood’s lamp-assisted dermoscopy suggests that ultraviolet illumination may represent a useful adjunctive tool to better visualize interface-related pigmentation changes in complex lesions, potentially improving recognition of subtle melanocytic components in routine clinical practice.

## 5. Conclusions

The integration of conventional dermoscopy with complementary techniques, including polarized dermoscopy and Wood’s lamp-assisted examination, may further support the assessment of complex seborrheic keratosis-associated lesions by improving evaluation of lesion architecture and interface dynamics. Although the clinical utility of these approaches requires further validation, increased awareness of interface-focused dermoscopic patterns may contribute to earlier recognition of clinically inconspicuous melanocytic proliferations arising in association with seborrheic keratosis.

These observations should be considered hypothesis-generating and warrant confirmation in larger prospective studies.

## Figures and Tables

**Table 1:** Dermoscopic interface features of melanoma–seborrheic keratosis collision tumors.

Study (Year)	Melanoma Pigmentation	SK Dermoscopic Morphology	Collision Configuration	Interface-Related Darkening of SK	Asymmetric SK Pigmentation
Birnie & Varma (2008) [13]	Pigmented	Not specified	Melanoma-dominant	Yes	Yes
Fikrle et al. (2021) [11]	Pigmented	Cerebriform pattern	Half-to-half	Yes	Yes
DeFazio et al. (2012) [12]	Pigmented	Cerebriform pattern	SK-dominant	Yes	Yes
Zaballos et al. (2022) [10]	Pigmented	Comedo-like openings and milia-like cysts	SK-dominant	Yes	Yes
Blum et al. (2017) [14]	Hypopigmented	Seborrheic keratosis with sulci/crypts	Half-to-half	No	Yes

## Data Availability

Data sharing is not applicable to this article, as no new datasets were generated. The scoping review is based on previously published studies, and the illustrative case is presented for descriptive purposes only.

## Supplementary Materials

The following supporting information can be downloaded at: https://www.mdpi.com/article/10.3390/diagnostics16081226/s1, Table S1: Quality appraisal of the included studies according to predefined methodological criteria. Checklist S1: PRISMA 2020 checklist. Reference [34] is cited in the Supplementary Materials.

## Abbreviations

The following abbreviations are used in this manuscript:

SK	Seborrheic keratosis
LM	Lentigo maligna
LMM	Lentigo maligna melanoma
RCM	Reflectance confocal microscopy
PRISMA	Preferred Reporting Items for Systematic Reviews and Meta-Analyses
UV	Ultraviolet
nm	Nanometer

## References

[1] Kittler H., Pehamberger H., Wolff K., Binder M. (2002). Diagnostic accuracy of dermoscopy. Lancet Oncol..

[2] Vestergaard M.E., Macaskill P., Holt P.E., Menzies S.W. (2008). Dermoscopy Compared with Naked Eye Examination for the Diagnosis of Primary Melanoma: A Meta-Analysis of Studies Performed in a Clinical Setting. Br. J. Dermatol..

[3] Garbe C., Amaral T., Peris K., Hauschild A., Arenberger P., Basset-Seguin N., Bastholt L., Bataille V., Brochez L., del Marmol V. (2025). European Consensus-Based Interdisciplinary Guideline for Melanoma. Part 2: Treatment—Update 2024. Eur. J. Cancer.

[4] Scope A., Longo C. (2014). Recognizing the Benefits and Pitfalls of Reflectance Confocal Microscopy in Melanoma Diagnosis. Dermatol. Pract. Concept..

[5] Bulte C.A., Hoegler K.M., Khachemoune A. (2020). Collision Tumors: A Review of Their Types, Pathogenesis, and Diagnostic Challenges. Dermatol. Ther..

[6] Minagawa A. (2017). Dermoscopy–Pathology Relationship in Seborrheic Keratosis. J. Dermatol..

[7] Argenziano G., Albertini G., Castagnetti F., De Pace B., Di Lernia V., Longo C., Pellacani G., Piana S., Ricci C., Zalaudek I. (2012). Early Diagnosis of Melanoma: What Is the Impact of Dermoscopy?. Dermatol. Ther..

[8] Dyer J.M., Foy V.M. Revealing the Unseen: A Review of Wood’s Lamp in Dermatology—PubMed. https://pubmed.ncbi.nlm.nih.gov/35783566/.

[9] Tricco A.C., Lillie E., Zarin W., O’Brien K.K., Colquhoun H., Levac D., Moher D., Peters M.D.J., Horsley T., Weeks L. (2018). PRISMA Extension for Scoping Reviews (PRISMA-ScR): Checklist and Explanation. Ann. Intern. Med..

[10] Zaballos P., Álvarez Salafranca M., Medina C., Bañuls J., Puig S., Del Pozo L.J., Malvehy J., Karaarslan I.K., Thomas L., Landi C. (2022). The Usefulness of Dermoscopy for the Recognition of Malignant Collision Tumors. Dermatology.

[11] Fikrle T., Divisova B., Pizinger K. (2021). Clinical-Dermoscopic-Histopathological Correlations in Collision Skin Tumours. Indian J. Dermatol..

[12] DeFazio J., Zalaudek I., Busam K.J., Cota C., Marghoob A. (2012). Association between Melanocytic Neoplasms and Seborrheic Keratosis: More than a Coincidental Collision?. Dermatol. Pract. Concept..

[13] Birnie A.J., Varma S. (2008). A Dermatoscopically Diagnosed Collision Tumour: Malignant Melanoma Arising within a Seborrhoeic Keratosis. Clin. Exp. Dermatol..

[14] Blum A., Siggs G., Marghoob A. (2017). Collision Skin Lesions—Results of a Multicenter Study of the International Dermoscopy Society (IDS). Dermatol. Pract. Concept..

[15] Braun R.P., Rabinovitz H.S., Krischer J., Kreusch J., Oliviero M., Naldi L., Kopf A.W., Saurat J.H. (2002). Dermoscopy of pigmented seborrheic keratosis: A morphological study. Arch. Dermatol..

[16] Lallas K., Arceu M., Martinez G., Manoli S.M., Papageorgiou C., Ilieva A., Todorovska V., Vakirlis E., Sotiriou E., Ioannides D. (2022). Dermoscopic Predictors of Benignity and Malignancy in Equivocal Lesions Predominated by Blue Color. Dermatology.

[17] Kittler H., Guitera P., Riedl E., Avramidis M., Teban L., Fiebiger M., Weger R.A., Dawid M., Menzies S. (2006). Identification of clinically featureless incipient melanoma using sequential dermoscopy imaging. Arch. Dermatol..

[18] Delker S., Livingstone E., Schimming T., Schadendorf D., Griewank K.G. (2017). Melanoma Diagnosed in Lesions Previously Treated by Laser Therapy. J. Dermatol..

[19] Cabrioli C., Maione V., Arisi M., Perantoni M., Guasco Pisani E., Venturini M., Calzavara-Pinton P., Licata G. (2023). Surgical Margin Mapping for Lentigo Maligna and Lentigo Maligna Melanoma: Traditional Technique (Visual Inspection with Dermoscopy) versus Combined Paper Tape and Reflectance Confocal Microscopy Technique. Int. J. Dermatol..

[20] Pietkiewicz P., Navarrete-Dechent C., Togawa Y., Szlązak P., Salwowska N., Marghoob A.A., Leszczyńska-Pietkiewicz A., Errichetti E. (2024). Applications of Ultraviolet and Sub-Ultraviolet Dermatoscopy in Neoplastic and Non-Neoplastic Dermatoses: A Systematic Review. Dermatol. Ther..

[21] Aboud D.M.A., Gossman W. (2023). Wood’s Light.

[22] Kwaśny M., Stachnio P., Bombalska A. (2025). Application of Wood’s Lamp in Dermatological and Dental Photodiagnostics. Sensors.

[23] Lu Q.S., Chen X., Wang S., Xu S.S., Wu T., Jiang G., Guo L.S. (2020). Dermoscopy Combined with Wood Lamp, a Diagnostic Alternative for Five Pigmented Lesions on the Face: An Observational Study. Chin. Med. J..

[24] Navarro-Navarro I., Ortiz-Prieto A., Villegas-Romero I., Valenzuela-Ubiña S., Linares-Barrios M. (2022). Wood’s Lamp for Delineating Surgical Margins in Lentigo Maligna and Lentigo Maligna Melanoma. Actas Dermosifiliogr..

[25] Green K.J., Pokorny J., Jarrell B. (2024). Dangerous Liaisons: Loss of Keratinocyte Control over Melanocytes in Melanomagenesis. BioEssays.

[26] Barthelmann S., Butsch F., Lang B.M., Stege H., Großmann B., Schepler H., Grabbe S. (2023). Seborrheic Keratosis. JDDG J. Dtsch. Dermatol. Ges..

[27] Heidenreich B., Denisova E., Rachakonda S., Sanmartin O., Dereani T., Hosen I., Nagore E., Kumar R. (2017). Genetic alterations in seborrheic keratoses. Oncotarget.

[28] Hafner C., López-Knowles E., Luis N.M., Toll A., Baselga E., Fernández-Casado A., Hernández S., Ribé A., Mentzel T., Stoehr R. (2007). Oncogenic PIK3CA mutations occur in epidermal nevi and seborrheic keratoses with a characteristic mutation pattern. Proc. Natl. Acad. Sci. USA.

[29] Hafner C., Vogt T., Landthaler M., Müsebeck J. (2008). Somatic FGFR3 and PIK3CA Mutations Are Present in Familial Seborrhoeic Keratoses. Br. J. Dermatol..

[30] Upadhyay P.R., Ho T., Abdel-Malek Z.A. (2021). Participation of Keratinocyte- and Fibroblast-Derived Factors in Melanocyte Homeostasis, the Response to UV, and Pigmentary Disorders. Pigment. Cell Melanoma Res..

[31] Cui Y.Z., Man X.Y. (2023). Biology of Melanocytes in Mammals. Front. Cell Dev. Biol..

[32] da Silva C.N., Miot H.A., Grassi T.F., Dias-Melício L.A., Santos L., Espósito A.C.C. (2023). Expression of Endothelin-1, Endothelin Receptor-A, and Endothelin Receptor-B in Facial Melasma Compared to Adjacent Skin. Clin. Cosmet. Investig. Dermatol..

[33] Pham D.M., Guhan S., Tsao H. (2020). Kit and Melanoma: Biological Insights and Clinical Implications. Yonsei Med. J..

[34] Page M.J., McKenzie J.E., Bossuyt P.M., Boutron I., Hoffmann T.C., Mulrow C.D., Shamseer L., Tetzlaff J.M., Akl E.A., Brennan S.E. (2021). The PRISMA 2020 statement: An updated guideline for reporting systematic reviews. BMJ.

