# Supplementary material for: Dermoscopic Interface Features in Melanoma–Seborrheic Keratosis Collision Tumors: A Scoping Review with an Illustrative Case Report on Wood’s Lamp Dermoscopy
Source: Diagnostics (Basel). 2026 Apr 20;16(8):1226. doi: 10.3390/diagnostics16081226 (PMC13114341; doi:10.3390/diagnostics16081226)
Supplement: Supplementary file 1 [file diagnostics-16-01226-s001.zip › Table S1 quality appraisal.pdf]

Supplementary Table S1. Quality appraisal of included studies

Study	Study type	Cases clearly described	Dermoscopic documentation available	Histopathology confirms melanoma + SK	Limitations stated
Birnie & Varma 2008	Case report	Yes	Yes	Yes	No
Association between melanocytic neoplasms and seborrheic keratosis – case series	Case series	Yes	Yes	Yes	Unclear
Fikrle et al. 2021 (melanoma–SK subset)	Case series	Yes	Yes	Yes	Yes
Zaballos et al. 2022 (melanoma–SK subset)	Case series	Yes	Yes	Yes	Yes
